# Limb and trunk accelerometer data collected with wearable sensors from subjects with Parkinson’s disease

**DOI:** 10.1038/s41597-021-00831-z

**Published:** 2021-02-05

**Authors:** Gloria Vergara-Diaz, Jean-Francois Daneault, Federico Parisi, Chen Admati, Christina Alfonso, Matilde Bertoli, Edoardo Bonizzoni, Gabriela Ferreira Carvalho, Gianluca Costante, Eric Eduardo Fabara, Naama Fixler, Fatemah Noushin Golabchi, John Growdon, Stefano Sapienza, Phil Snyder, Shahar Shpigelman, Lewis Sudarsky, Margaret Daeschler, Lauren Bataille, Solveig K. Sieberts, Larsson Omberg, Steven Moore, Paolo Bonato

**Affiliations:** 1 Department of Physical Medicine and Rehabilitation, Harvard Medical School, Spaulding Rehabilitation Hospital, Boston, Massachusetts USA; 2 Department of Rehabilitation and Movement Sciences, Rutgers University, Newark, New Jersey USA; 3 Intel Corporation, IT Advanced Analytics, HaMerkaz, Israel; 4 Department of Neurology, Icahn School of Medicine at Mount Sinai, New York, New York USA; 5 Department of Neurology, Harvard Medical School, Massachusetts General Hospital, Boston, Massachusetts USA; 6 Sage Bionetworks, Seattle, Washington 98109 USA; 7 Department of Neurology, Harvard Medical School, Brigham and Women’s Hospital, Boston, Massachusetts USA; 8 Michael J Fox Foundation, New York, New York USA; 9 School of Engineering and Technology, Central Queensland University, Rockhampton, Australia; 10 Wyss Institute for Biologically Inspired Engineering, Harvard University, Cambridge, Massachusetts USA

**Keywords:** Parkinson's disease, Parkinson's disease

## Abstract

Parkinson’s disease (PD) is a neurodegenerative disorder characterized by motor and non-motor symptoms. Dyskinesia and motor fluctuations are complications of PD medications. An objective measure of on/off time with/without dyskinesia has been sought for some time because it would facilitate the titration of medications. The objective of the dataset herein presented is to assess if wearable sensor data can be used to generate accurate estimates of limb-specific symptom severity. Nineteen subjects with PD experiencing motor fluctuations were asked to wear a total of five wearable sensors on both forearms and shanks, as well as on the lower back. Accelerometer data was collected for four days, including two laboratory visits lasting 3 to 4 hours each while the remainder of the time was spent at home and in the community. During the laboratory visits, subjects performed a battery of motor tasks while clinicians rated limb-specific symptom severity. At home, subjects were instructed to use a smartphone app that guided the periodic performance of a set of motor tasks.

## Abstract (summary)

**Table:** 

Measurement(s)	body movement coordination trait • Movement Disorder Society Unified Parkinson’s Disease Rating Scale Questionnaire • Medication • motor coordination/balance trait • sleep pattern • MDS-UPDRS Tasks and Simulated Activities of Daily Living (in-clinic) • Activity of Daily Living
Technology Type(s)	Accelerometer • body movement/behavior method • Clinical Observation • smartphone • Subject Diary
Factor Type(s)	age of patient • gender of patient • timing of medication intake
Sample Characteristic - Organism	Homo sapiens

Machine-accessible metadata file describing the reported data: 10.6084/m9.figshare.13574279

## Background & Summary

Parkinson’s disease (PD) is one of the most common neurodegenerative disorders, occurring in about 2% of the population over the age of sixty^1,2^. PD is primarily characterized by motor symptoms, such as resting tremor, rigidity, and bradykinesia, but is also associated with several non-motor symptoms^2,3^. Levodopa (L-dopa), a biosynthetic precursor of dopamine, is the gold standard treatment for people with PD^4^. As the disease progresses, dyskinesia and motor fluctuations are prominent complications of L-dopa treatment that affect the majority of individuals with PD^3^. Thus, a progressive loss in quality of life due to motor and non-motor symptoms, as well as treatment side-effects, is experienced by people with PD^5,6^.

The causes of motor fluctuations are not entirely clear^7^ and the tools currently available for monitoring and managing motor fluctuations are quite limited^8–11^. There continue to be significant efforts to develop therapeutic strategies that reduce motor complications and fluctuations (e.g. adjunct anti-parkinsonian medications, duodenal levodopa, deep brain stimulation (DBS), etc.)^12^ and a reliable, reproducible, and objective measure of on/off time with/without dyskinesia would likely help clinicians to assess the response to the intervention and facilitate modifications in the patients’ medication regimen.

In an accompanying manuscript^13^, we made available a dataset from twenty-eight subjects with PD that were recruited from two sites in which tri-axial accelerometer data was collected continuously using two commercially available smartwatch-like wrist-worn accelerometers (GeneActiv and Pebble) and a waist-worn smartphone during a period of four days. Herein, we describe and make available a unique dataset that was collected simultaneously in a subset of subjects using an additional set of sensors. The aim was to capture limb-specific fluctuations in symptom severity and motor states over the same 4 days as in the companion manuscript. This dataset was collected simultaneously (i.e. subjects wore all the sensors at the same time) in 19 of the subjects whose data is reported in the companion paper using the Sensing Health with Intelligence, Modularity, Mobility, and Experimental Reusability (Shimmer) 3 sensing platform. Using this dataset, one can observe continuous changes in limb-specific symptom severity during the day and across medication cycles. The dataset was captured using wearable sensors both in a laboratory setting (with ground truth labels of symptom severity and scripted activities being performed in the laboratory) and in the home setting (with a set of known points in time when subjects were guided by a smartphone app to perform scripted tasks). The dataset herein presented complements the one presented in the companion manuscript that focused on a minimum set of consumer-grade sensors (i.e. a Pebble smartwatch, a GeneActiv wearable sensor, and a Samsung smartphone). In contrast with the dataset presented in the companion manuscript, the one herein presented contains data collected from sensors located on each lower-limb, each upper-limb, and on the lower back. In addition, the data collected in the home and community setting includes timestamps corresponding to instances when a smartphone app was used to instruct subjects to perform a set of motor tasks. These motor tasks were performed both in the laboratory and in the home. Hence, one can envision using the data collected in the laboratory to develop algorithms applicable to the data collected in the home during the performance of these scripted motor activities. This dataset also complements other available wearable sensor datasets such as the mPower dataset^14^, which was not collected with specific focus on individuals experiencing motor fluctuations and used solely a smartphone to gather data in the home and community settings, including questionnaires, sensor data related to gait and balance impairments, and data collected during the performance of standardized tasks to assess the effects of symptoms such bradykinesia and tremor on movements performed using distal body segments. Finally, the dataset presented in this manuscript complements the Daphnet Freezing of Gait Dataset^15^, which consists of accelerometer data collected in the laboratory setting using wearable sensors placed on the lower limbs and trunk in subjects with PD experiencing freezing of gait.

It is well known that symptom severity in people with PD can fluctuate and can differ among limbs^3,7^. It has been suggested that a minimum of one wearable sensor per limb is required in order to obtain limb-specific symptom severity scores^16^. While it has been proposed that the symptoms of PD may become more symmetric between the dominant and non-dominant limbs over time, this does not occur for all individuals^17^. Furthermore, discrepancies in symptom severity between upper and lower limbs are routinely observed^18^. As such, we opted to have subjects don five wearable sensors (i.e. one on each limb and one at the lower back) to capture motor behaviors enabling the estimation of limb-specific symptom severity. It is worth mentioning that the motor examination of the MDS-UPDRS is meant to be performed by observing symptoms on a segment-by-segment basis thus providing limb-specific scores for rigidity, bradykinesia and tremor. For this reason, having a sensor on each limb enables matching the limb-specific clinical scores with estimates derived from the sensor data.

Subjects recruited in the study came to the Motion Analysis Laboratory at Spaulding Rehabilitation Hospital (Boston, MA) while on their usual medication schedule on Day 1. Shimmer 3 sensors were placed at the level of the fifth lumbar vertebrae (L5) as well as on both forearms and shanks. Subjects were asked to perform all the items of part III of the MDS-UPDRS^19^ once. Then, they were asked to complete a battery of motor tasks lasting about 20 minutes that included selected motor tasks taken from part III of the MDS-UPDRS, and some activities of daily living. This battery of tasks was repeated at 30-minute intervals, typically a total of 6 times. Once the data collection in the laboratory was completed, subjects went home while wearing the sensors. During the next two days, they were instructed to carry out their usual activities. In addition, they were asked to perform, at given times of those two days, a short set of motor tasks consisting of three items of section III of the MDS-UPDRS (i.e. sitting quietly, finger-to-nose, and pronation-supination). On Day 3, subjects were asked to withhold their medication/s overnight in order to come to the laboratory on Day 4 in a practically defined off state. A portion of the same procedures that were performed on Day 1 were carried out once again on Day 4. After the data collection, subjects doffed the sensors.

## Methods

### Participants

**Fig. 1:**
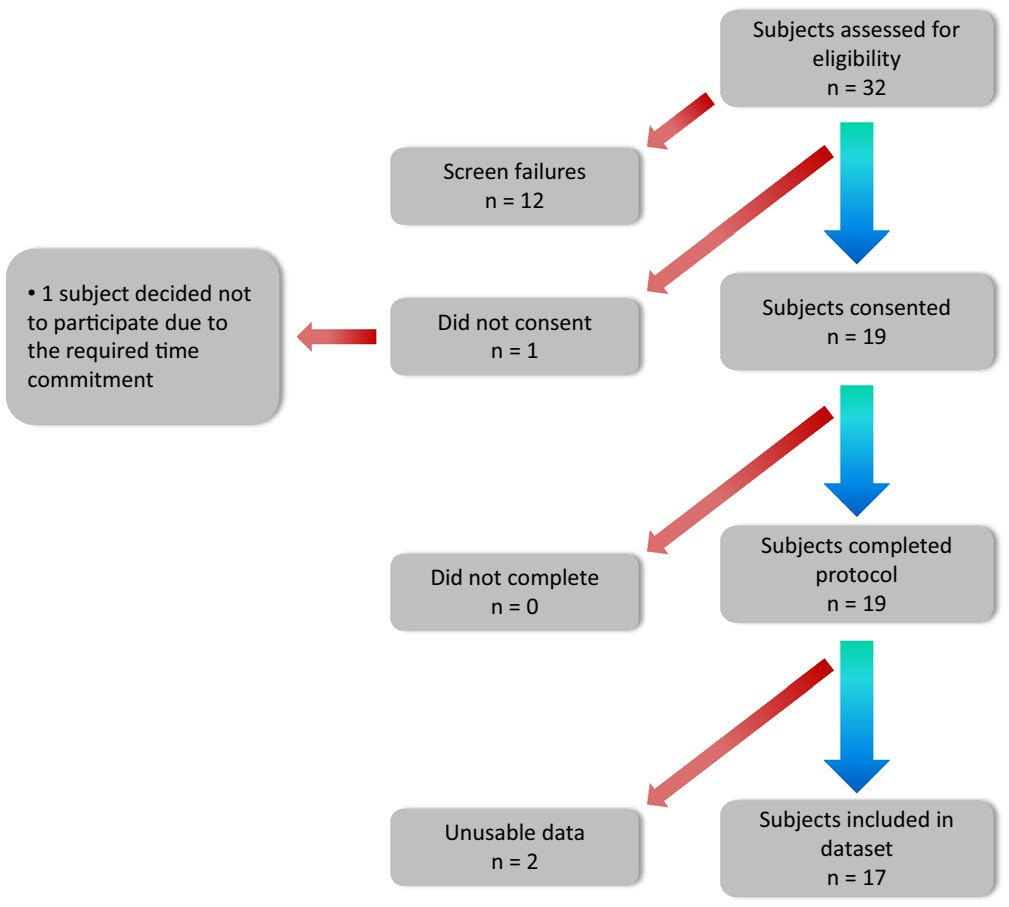
Flow diagram of participant onboarding in the study. The data from one subject was excluded from the dataset because he performed tasks that were slightly different from all other subjects. The data from another subject was excluded because a technical malfunction of the sensors led to a significant amount of data being lost.

A total of 19 participants with PD experiencing motor fluctuations were enrolled in the study. All subjects signed the informed consent form. Individuals were eligible if they were: community dwelling men and women between 30 and 80 years of age; diagnosed with idiopathic PD; taking levodopa; experiencing self-reported motor fluctuations and at least mild dyskinesia; and capable of using a smartphone. Individuals were excluded from the study for the following reasons: history of any major neurological condition (other than PD); and use of deep brain stimulation (DBS). The Institutional Review Board at Spaulding Rehabilitation Hospital approved this study (#2014P000847). Data collected from two individuals had to be excluded (Fig. 1). The first enrolled subject performed slightly different motor tasks than all other subjects. In fact, the protocol was modified to address issues that we experienced during the first data collection. For the other subject, we experienced technical issues with the sensors hence leading to a significant data loss. We therefore opted to exclude the datasets collected from these two subjects.

### Data collection

Subjects were asked to participate in a first study visit during Day 1 in an on-medication state, in a two day at-home data collection while maintaining a regular medication regimen, and in a final study visit for which subjects were instructed to come to the laboratory in a practically-defined off state. Both study visits were performed in the Motion Analysis Laboratory at Spaulding Rehabilitation Hospital. All study participants were asked to withhold antiparkinsonian medications for approximately 12 hours prior to the second laboratory visit and to take their medication/s right after completing the first battery of tasks.

**Fig. 2:**
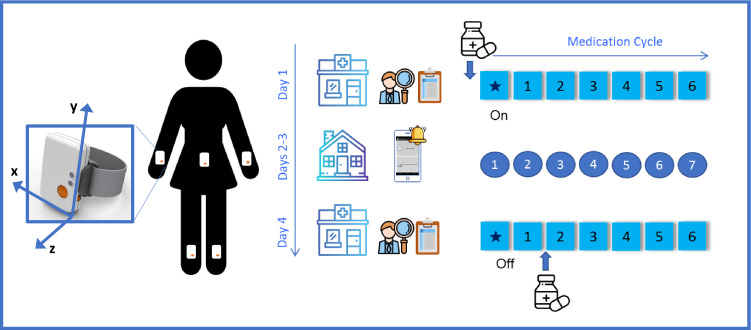
Overview of the Study Protocol. Study participants wore five Shimmer 3 sensors (reference axes are shown by the blue arrows in the inset) over four consecutive days. During Days 1 and 4 - when we recorded data in the laboratory - subjects were asked to performed part III of the MDS-UPDRS followed by a battery of tasks that were repeated 6 times. The * symbol represents the performance of the MDS-UPDRS. During Days 2 and 3 - when we recorded data in the home and community settings - subjects were asked to perform 7 times a short battery of tasks.

A schematic representation of the data collection procedures is shown in Fig. 2. Two participants deviated from the prescribed medication protocol: one subject (4_BOS) arrived in the off state to the hospital on Day 1 and in the on state on Day 4. One subject (3_BOS) had a medication intake before the beginning of the second laboratory visit (medication intake 4 hours before the laboratory visit). A summary of all the available data is shown in Table 1 (Sensor Data – Part II in Table 2^20^, Task Scores – Part II in Table 3^20^, Metadata of Patient Onboarding in Table 4^20^, Metadata of Laboratory Visits in Table 5^20^, Subjects Diary in Table 6^20^, Medication Diary in Table 7^20^, Sleep Diary in Table 8^20^, Home Tasks in Table 9^20^, UPDRS Responses in Table 10^20^, Total Duration and Percentage of Valid Data in Table 11, Detailed Duration and Percentage of Valid Data in Online-only Table 1, and Sensor Failure Notes in Table 12).

**Table 1:** Data available.

Task name	Type of task and schedule	Table ^Reference^
Sensor Data – Part II	Activity – Four days	Table 2^20^
Task Scores – Part II	Assessment - Twice	Table 3^20^
Metadata of Patient Onboarding	Survey – Once	Table 4^20^
Metadata of Laboratory Visits	Survey – Twice	Table 5^20^
Subjects Diary	Survey – Twice	Table 6^20^
Medication Diary	Survey - Once	Table 7^20^
Sleep Diary	Survey - Once	Table 8^20^
Home Tasks	Activity – Two days	Table 9^20^
UPDRS Responses	Assessment - Twice	Table 10^20^
Total Duration and Percentage of Valid Data	Not Applicable	Table 11
Detailed Duration and Percentage of Valid Data	Not Applicable	Online-only Table 1
Sensor Failure Notes	Not Applicable	Table 12

**Table 2:** Sensors Data – Part II.

Variable name	Variable details
subject_id	Alphanumeric
device	‘Shimmer’
device_position	One of {‘Back’, ‘RightUpperLimb’, ‘LeftUpperLimb’, ‘RightLowerLimb’, ‘LeftLowerLimb’}
participant_day	Integer
timestamp_start	Real number
timestamp_end	Real number
source_file	Hyperlink
data_file_handle_id	Hyperlink

**Table 3:** Task Scores – Part II.

Variable name	Variable details
subject_id	Alphanumeric
visit	Integer
session	Integer
task_id	Integer
task_code	One of {‘stndg’, ‘wlkgs’, ‘wlkgc’, ‘strsu’, ‘strsd’, ‘wlkgp’, ‘drawg’, ‘ftnr’, ‘ftnl’, ‘ramr’, ‘raml’, ‘ststd’, ‘typng’, ‘ntblt’, ‘drnkg’, ‘orgpa’, ‘fldng’, ‘sittg’}
repetition	One of {‘1’, ‘2’}
timestamp_start	Real number
timestamp_end	Real number
phenotype	One of {‘tremor’, ‘dyskinesia’, ‘bradykinesia’}
body_region	One of {‘RightUpperLimb’, ‘LeftUpperLimb’, ‘RightLowerLimb’, ‘LeftLowerLimb’}
score	One of {‘Yes’, ‘No’, ‘NotApplicable’, ‘0’, ‘1’, ‘2’, ‘3’, ‘4’}

**Table 4:** Metadata of Patient Onboarding.

Variable name	Variable details
subject_id	Alphanumeric
cohort	PD
gender	One of {‘Male’, ‘Female’}
birth_year	Integer
dominant_hand	One of {‘Right’, ‘Left’}
upper_limb_length	Real number
upper_arm_length	Real number
lower_arm_length	Real number
lower_limb_length	Real number
thigh_length	Real number
shank_length	Real number
height	Real number
weight	Real number
visit_date	Date
diagnosis_day	Integer
diagnosis_month	Integer
diagnosis_year	Integer
pd_most_affected_side	One of {‘Right’, ‘Left’, ‘Bilateral’}
gait_impediments	Boolean
posture_instability	Boolean
tremor	Boolean
bradykinesia	Boolean
disrupted_sleep	Boolean
freeze_of_gait	Boolean
dyskinesia	Boolean
rigidity	Boolean
other_symptoms	Text
last_levodopa_dose_timestamp	Integer
regular_medication	Text
geneactive_num	Integer
pebble_num	Alphanumeric
geneactive_hand	One of {‘Right’, ‘Left’}
pebble_hand	One of {‘Right’, ‘Left’}
smartphone_location	One of {‘Right’, ‘Left’}
recording_start	Time
recording_end	Time
timezone	Text
updrs_time	Time
updrs_score_p1	Integer
updrs_score_p2	Integer
updrs_score_p3	Integer
updrs_score_p4	Integer
h_and_y_score	Integer
updrs_second_visit_time	Time
updrs_second_visit_score_p3	Integer

**Table 5:** Metadata of Laboratory Visits.

Variable name	Variable details
subject_id	Alphanumeric
visit	One of {‘1’, ‘2’}
clinical_assessment_timestamp	Integer
medication_intake_timestamp	Integer
medication_name	Text
medication_dosage	Text
timezone	Text
second_medication_intake_timestamp	Integer
stopwatch_start_timestamp	Integer
fox_insight_app_start_timestamp	Integer
geneactiv_start_timestamp	Integer
general_comments	Text

**Table 6:** Subjects Diary.

Variable name	Variable details
subject_id	Alphanumeric
participant_day	One of {‘2’, ‘3’}
session_label	Text
session_number	Integer
off	Boolean
dyskinesia	Boolean
troublesome_dyskinesia	Boolean
tremor	Boolean
freeze_of_gait	Boolean
slowness_of_movement	Boolean
comments	Text

**Table 7:** Medication Diary.

Variable name	Variable details
subject_id	Alphanumeric
med_id	Integer
med_timestamp_date	Date
med_timestamp_hour	Time
timestamp	Integer
pd_related_medications	Text
other_medications	Text

**Table 8:** Sleep Diary.

variable name	Variable details
subject_id	Alphanumeric
entry_id	Integer
sleep_event_date	Date
sleep_event_hour	Time
timestamp	Integer
sleep_event_type	One of {‘fall_asleep_time’, ‘wake_up_time’}

**Table 9:** Home Tasks.

Variable name	Variable details
subject_id	Alphanumeric
participant_day	One of {‘2’, ‘3’}
session	Integer in the range [1–10]
task_code	One of {‘ftnr’, ‘ftnl’, ‘ramr’, ‘raml’, ‘sittg’}
timestamp_start	Real number
timestamp_end	Real number
seconds_since_last_med_intake	Real number

**Table 10:** UPDRS Responses.

Variable name	Variable details
subject_id	Alphanumeric
visit	One of {‘1’, ‘2’}
item_code	Alphanumeric
item_desc	Text
item_value	Integer or NA If **is_scored** is true one of {0, 1, 2, 3, 4} (mapping to {‘Normal’, ‘Slight’, ‘Mild’, ‘Moderate’, ‘Severe’})
is_scored	Boolean

**Table 11:** Total Duration and Percentage of Valid Data- RUL: Right Upper Limb, LUL: Left Upper Limb; RLL: Right Lower Limb; LLL: Left Lower Limb; BK: Back.

Subject ID	Duration (hours)	% valid Data				
		RUL	LUL	RLL	LLL	BK
3_BOS	75.43	100%	100%	100%	100%	100%
4_BOS	75.62	100%	100%	100%	100%	68%
5_BOS	76.79	100%	100%	100%	100%	100%
6_BOS	74.79	100%	100%	44.2%	100%	100%
7_BOS	75.02	100%	100%	100%	100%	100%
8_BOS	75.45	100%	100%	100%	100%	100%
9_BOS	75.53	100%	100%	100%	100%	7%
10_BOS	74.21	100%	100%	100%	100%	100%
11_BOS	76.26	64.6%	100%	100%	100%	29.7%
12_BOS	77.18	100%	100%	100%	100%	100%
13_BOS	76.26	100%	100%	100%	100%	100%^1^
14_BOS	75.89	100%	100%	100%	3.5%	61%
15_BOS	74.93	100%	100%	100%	100%	100%
16_BOS	76.17	100%	100%	100%	100%	100%
17_BOS	77.48	100%	100%	100%	100%	100%
18_BOS	75.06	100%	100%	100%	100%	100%
19_BOS	75.51	100%	100%	100%	100%	100%

^1^No missing data but the subject took the sensor off at home (2015/06/22 17:45:30). The sensor was placed back by the research staff on Day 4 (2015/06/25 7:48:30), before the beginning of the second visit in the laboratory.

**Table 12:** Sensor Failure Notes.

Subject ID	Sensor Failure Note 1	Sensor Failure Note 2
4_BOS	Back sensor ran out of battery - partial data on Day 4	
6_BOS	Left Lower Limb sensor ran out of battery - partial data on Day 2, no data on Day 3–4	
9_BOS	Back sensor was stopped by mistake by the subject - partial data on Day 1, no data on Day 2-3-4	
11_BOS	Right Upper Limb sensor had technical problems - partial data on Day 2, no data Day 3–4	Back sensor had technical problems - partial data on Day 2, no data on Day 3–4
13_BOS	Back sensor was taken off by the subject at home (2015/06/22 17:45:30)	Back sensor placed back in the laboratory on Day 4 (2015/06/25 7:48:30)
14_BOS	Left Lowe Limb sensor was stopped by mistake by the subject - partial data on Day 1, no data on day 2-3-4	Back sensor run out of battery - partial data on Day 3, no data on Day 4

After signing the consent form, demographic and medical history data as well as height and weight of the subjects were recorded (Table 4) ^20^. MDS-UPDRS sections I, II and IV were also completed (Table 4) ^20^.

**Fig. 3:**
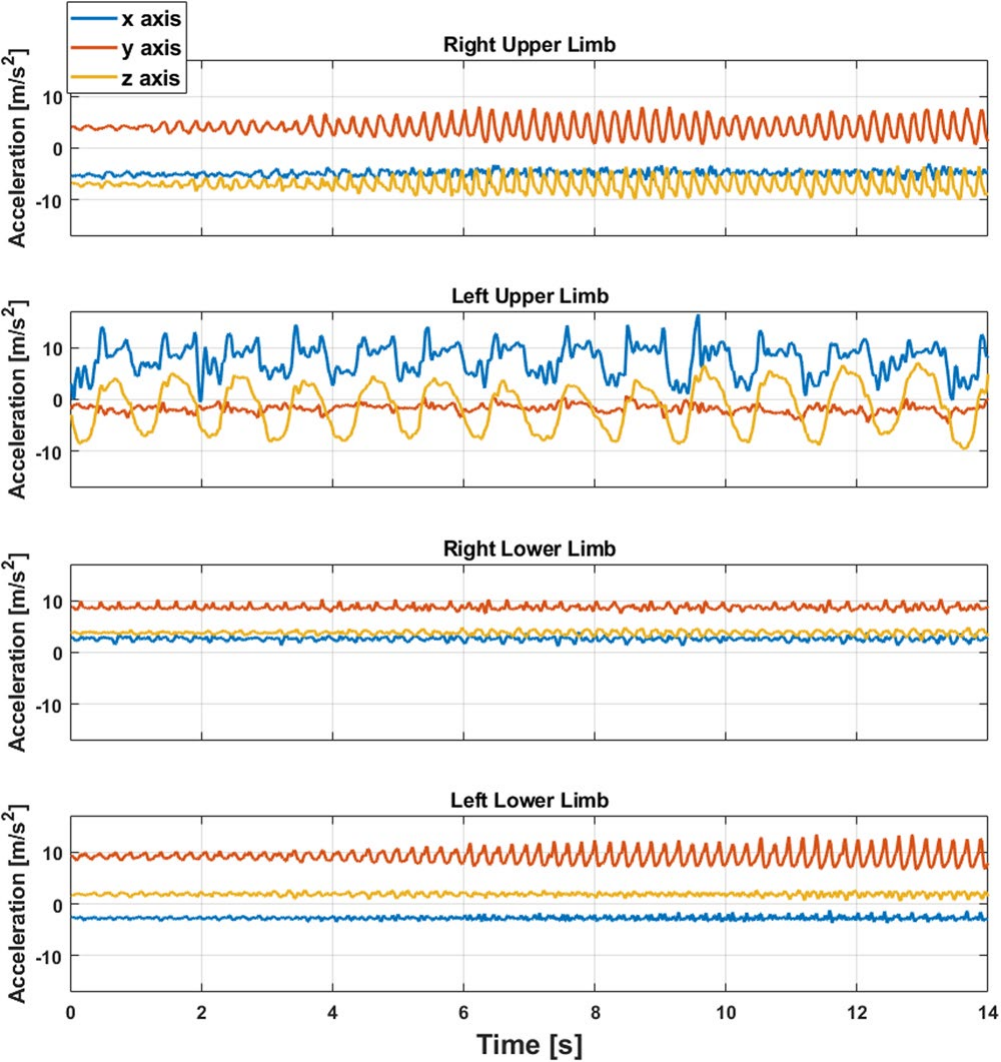
Example of accelerometer collected data during the performance of the alternate hand movement task. In this example, the subject performed the task with the left arm while all other limbs were at rest. Each of the sub-plots illustrates the axis-specific acceleration data (blue – x-axis; orange – y-axis; and yellow – z-axis). Note that the data is provided in a local coordinate frame.

The sensors used were Shimmer 3 units (Shimmer Research, Dublin, Ireland). Subjects were asked to don a total of five Shimmer 3 units, one on each forearm, one on each shank, and one on the lower back (L5). Some subjects reported that the lower back sensor was uncomfortable while sleeping. In one case (13_BOS), the subject removed the sensor to sleep more comfortably. The sensor was placed back by the study staff at the beginning of the second study visit on Day 4. 3D acceleration data was collected at 51.2 Hz from each Shimmer 3 sensor over four consecutive days (Table 2) ^20^. An example of the accelerometer data gathered during one task is shown in Fig. 3. An additional sensor was used to collect timestamps associated with the start and end times of the MDS-UPDRS and as reference sensor for the temporal synchronization of all the accelerometer data (see details in the Data Pre-processing subsection). Additional timestamps were associated with each motor task performed in the laboratory (Table 3) ^20^. All the sensors were shaken simultaneously at the beginning and at the end of the data collections to enable *a posteriori* synchronization of the time series.

PD motor symptom severity was assessed in the laboratory on Day 1 and Day 4 using the MDS-UPDRS motor examination subscale (section III) (Tables 4 and 10) ^20^. Subsequently, subjects performed the following battery of motor tasks: standing quietly for 30 seconds (stndg); walking in a straight line for 30 seconds (wlkgs); walking in a straight line for 30 seconds while counting backwards aloud (wlkgc); walking up the stairs (strsu); walking down the stairs (strsd); walking through a narrow corridor six times (wlkgp); finger-to-nose for 15 seconds (repeated twice with each arm) (ftnr1, ftnl1, ftnr2, ftnl2); rapid alternating hand movements for 15 seconds (repeated twice with each arm) (ramr1, raml1, ramr2, raml2); sit to stand repeated three times (ststd); drawing a spiral (drawg); typing on a keyboard for 30 seconds (typng); assembling ten nuts and bolts twice (ntblt); opening a bottle; pouring water and pretending to drink three times (drnkg); organizing sheets of paper in a folder twice (orgpa); folding a towel on a table three times while standing (fldng); and sitting quietly for 30 seconds (sittg). Except for the tasks where subjects had to walk up or down the stairs, the battery of motor task (20 of them) was repeated every 30 minutes, for a total of 6 repetitions (Table 5) ^20^. A clinician that was trained and certified to score the MDS-UPDRS provided limb-specific scores of symptom severity for all the repetitions of each task for tremor, dyskinesia, and bradykinesia (Table 3) ^20^. Tremor and dyskinesia severity scores ranged from 0–4. The presence or absence of upper- and lower-limb bradykinesia was evaluated for all tasks (yes/no), except for alternating hand movement where a severity score ranging from 0–4 based on the related MDS-UPDRS item was provided.

Once the laboratory data collection was completed, subjects went home while wearing the sensors. Subjects wore the five sensors at home for two complete days while they performed their usual activities and took their regularly scheduled medication/s. In addition, individuals were asked to perform 7 repetitions of a short battery of motor tasks every 30 minutes during one medication cycle, each day, while being guided through these tasks by a custom-designed smartphone app. The tasks included alternating hand movements for 30 seconds (once with each arm), finger-to-nose for 30 seconds (once with each arm) and sitting quietly for 30 seconds. The app was developed by our team to provide reminders to study participants to perform the activities at 30-minute intervals during one of their medication cycles as well as to collect the start and end time of each of the tasks (Table 9) ^20^. Subjects were also asked to complete a paper-based diary to report their symptoms (Table 6) ^20^, medication intake times and doses (Table 7) ^20^, and the time they went to sleep/woke up (Table 8) ^20^.

During the second laboratory visit, subjects underwent an evaluation of their motor symptoms using part III of the MDS-UPDRS followed by 6 repetitions of the battery of motor tasks performed on Day 1. During this second laboratory visit (Day 4), subjects were asked to come in a practically-defined off state. They then performed the first repetition of motor tasks in their off state and subsequently took their regularly scheduled morning medication/s. Subjects then completed 5 repetitions of the battery of motor tasks. We did so to enhance the symptom variability observed during medication cycles. The same trained clinician provided symptom severity scores for tremor, dyskinesia and bradykinesia for all repetitions of all tasks. Once data collection was completed, sensors were removed from the subjects.

### Data Pre-processing

The raw sensors data from the 5 shimmer sensors worn by the subjects and the additional reference sensor kept in the laboratory were pre-processed in order to achieve the following objectives:

1. Identify intervals with missing data in the raw signals
2. Resample the time series at the sampling rate of 50 Hz
3. Temporally align the signals from the different sensors

The reference device was equipped with a “push button” (generating digital pulses) to mark the time instances associated to the beginning and the end of each motor task in the laboratory. In addition, this sensor recorded accelerometer data for the entire duration of the data collection (it was left in the laboratory while acquiring continuous data) and it was used as reference device for the time alignment procedure described below.

All the preprocessing and alignment procedures were performed using custom-designed MATLAB (Mathworks, Natick, MA) code.

The raw data generated by all Shimmer devices were first processed by replacing gaps in the time series due to missing data with sequences of NaN’s (Not-a-Number’s, i.e., not valid values), according to the original sampling rate of each device (51.2 Hz). This procedure allowed us to obtain time series for the entire duration of the data collection, with time vectors containing only increasing values and acceleration signals including both valid and invalid data (i.e. usable and missing values). The time series obtained for each sensor were then resampled using a linear interpolation method to obtain a sampling rate equal to 50 Hz. Subsequently, the resampled signals were temporally aligned by exploiting a simultaneous physical “shake” of all the devices that was done at the beginning of the first session (Day 1) and at the end of the last session (Day 4) in the clinic. The “shake” event consisted in intense upward/downward movements of all devices held together. This was done by a member of the research staff in the laboratory. The event was associated with an easily distinguishable pattern in the accelerometer time series of each device, which enabled the extraction of temporal offsets between the reference Shimmer device and the other five Shimmer sensors worn by the subjects. The temporal offsets were estimated using a cross-correlation based technique. Since the internal clocks of the devices were subject to drift, the offsets on Day 1 could be slightly different from those on Day 4. In order to address this issue, the magnitude of the drift was computed from the difference between the offsets on Day 1 and Day 4 for the non-reference devices. Then, the drift was removed under the assumption that it developed linearly during the entire data collection. The drift-free time series were obtained by removing this linear trend from their time axis. Finally, the time alignment between all devices was achieved by shifting the time vectors of the non-reference devices by the offsets computed for Day 1. Although the clock drift was likely influenced by many factors, such as environmental temperature, we deemed appropriate to assume it being linear. We verified this assumption by visual inspection of the raw accelerometer signals in correspondence of the physical shake events. The error in the temporal alignment between sensors was consistently below 100 ms and hence considered negligible. The aligned time series were then saved on Synapse.org. The accelerometer data from the “push-button” Shimmer device was not used for any other purpose than the temporal alignment and was not posted on the data repository platform. It is worth noting that the synchronized digital pulse signal generated by the “push-button” device was used to determine the starting and ending time of the motor tasks performed by the subjects in the laboratory.

### Dataset descriptive statistics

**Fig. 4:**
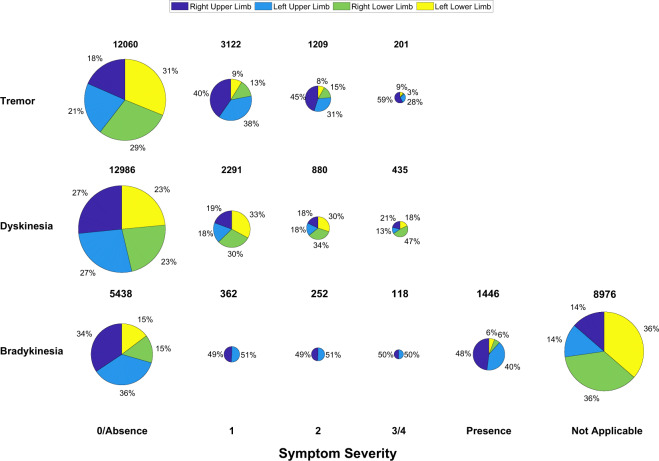
Pie chart representation of the distribution of the clinical scores assigned to the motor tasks performed during the laboratory visits for: (**a**) tremor, (**b**) dyskinesia, and (**c**) bradykinesia.

A total of 4,148 tasks were performed during the two laboratory visits by the 17 subjects whose data is part of the shared dataset. The distributions of the clinical scores for all the motor tasks performed during the laboratory visits are shown in Fig. 4. The number of instances for both the upper limbs and the lower limbs (combined) contributing to the total number of clinical scores for each severity or presence/absence of symptoms is provided.

Table 11 shows the total duration and the percentage of valid data for each sensing device and for each subject across the entire data collection period. Detailed information on the duration and the percentage of valid data for the two laboratory visits and the at-home period are provided in Online-only Table 1.

Table 12 shows sensor failure notes related to the data collections.

## Data Records

De-identified study data, consisting of questionnaire responses and Shimmers sensor data, were exported to Synapse. Synapse was developed and is operated by Sage Bionetworks. Synapse is a general-purpose data and analysis sharing service where members can work collaboratively, analyze data, share insights and have attributions and provenance of those insights to share with others.

A total of 19 subjects consented to participate in the study and completed the data collection procedures. For 17 subjects, we obtained data that could be utilized for analysis and hence shared. The data from two subjects was discarded. One of these subjects performed slightly different motor tasks from all other subjects. For the second subject, we experienced technical issues that led to the loss of a significant portion of the data.

All coded datasets are stored and accessible via the Synapse platform with associated metadata and documentation (10.7303/syn20681023) ^20^.

## Technical Validation

The data provided herein was collected using devices with proprietary technical validation. Hence, we do not provide test-retest nor other technical validation datasets. However, others have reported technical validation data for the sensors utilized in the study^21,22^. All the data was visually inspected by trained research staff.

## Usage Notes

Researchers who are interested in accessing the data need to complete the following steps:

1. Have a Synapse account (https://synapse.org)
2. Have their Synapse User Profile validated by the Synapse Access and Compliance Team (ACT)
3. Become a Synapse Certified user
4. Submit an Intended Data Use statement
5. Agree to the Conditions for Use associated with each data source (see DOIs for each data source)

While certain data types may have additional Conditions for Use (e.g. clinical scale copyrights), the overarching Conditions for Use are as follows:

- You confirm that you will not attempt to re-identify research participants for any reason, including for re-identification theory research.
- You reaffirm your commitment to the Synapse Awareness and Ethics Pledge.
- You agree to abide by the guiding principles for responsible research use and data handling as described in the Synapse Governance documents.
- You commit to keeping the data confidential and secure.
- You agree to use the data exclusively as described in your submitted Intended Data Use statement.
- You understand that the data may not be used for commercial advertisement or to re-contact research participants.
- You agree to report any misuse or data release, intentional or inadvertent to the ACT within 5 business days by emailing act@sagebase.org.
- You agree to publish findings in open access publications.
- You promise to acknowledge the L-dopa study investigators in all publications and presentations resulting from using the data as follows: “These data were part of the L-dopa study funded by the Michael J Fox Foundation”.

### Download the data

The data are stored in the Synapse data repository and can be accessed with different modalities:

1. Web-based download: the user can individually download each file directly from the web browser;
2. Python, R, and command line clients;
3. REST API.

Additional information and code examples about the data access procedures for this specific dataset can be found at https://www.synapse.org/#!Synapse:syn20681023/wiki. Generic documentation about the APIs for interacting with Synapse data repositories are available at https://docs.synapse.org/articles/api_documentation.html.

## Data Availability

The only data processing procedures that we performed on the dataset were the ones described above. The first procedure was carried out to temporally align the data collected using different sensors. The second procedure was carried out to obtain an evenly-sampled timeseries.

## Online-only Table

**Online-only Table 1:** Detailed Duration and Percentage of Valid Data- RUL: Right Upper Limb, LUL: Left Upper Limb; RLL: Right Lower Limb; LLL: Left Lower Limb; BK: Back.

Subject ID	Visit 1 Laboratory (Day 1)						Visit 2 Laboratory (Day 4)						Home					
	Duration (hours)	% Valid Data					Duration (hours)	% Valid Data					Duration (hours)	% Valid Data				
		RUL	LUL	RLL	LLL	BK		RUL	LUL	RLL	LLL	BK		RUL	LUL	RLL	LLL	BK
3_BOS	4.34	100%	100%	100%	100%	100%	3.35	100%	100%	100%	100%	100%	67.74	100%	100%	100%	100%	100%
4_BOS	4.54	100%	100%	100%	100%	100%	0.93	100%	100%	100%	100%	0%	68.14	100%	100%	100%	100%	68.9%
5_BOS	4.47	100%	100%	100%	100%	100%	3.95	100%	100%	100%	100%	100%	68.37	100%	100%	100%	100%	100%
6_BOS	3.78	100%	100%	100%	100%	100%	2.89	100%	100%	0%	100%	100%	68.12	100%	100%	43%	100%	100%
7_BOS	3.99	100%	100%	100%	100%	100%	3.07	100%	100%	100%	100%	100%	67.96	100%	100%	100%	100%	100%
8_BOS	4.09	100%	100%	100%	100%	100%	2.81	100%	100%	100%	100%	100%	68.55	100%	100%	100%	100%	100%
9_BOS	3.83	100%	100%	100%	100%	100%	3.23	100%	100%	100%	100%	0%	68.48	100%	100%	100%	100%	2.1%
10_BOS	5.10	100%	100%	100%	100%	100%	3.25	100%	100%	100%	100%	100%	65.86	100%	100%	100%	100%	100%
11_BOS	4.44	100%	100%	100%	100%	100%	3.93	0%	100%	100%	100%	0%	67.88	66.1%	100%	100%	100%	26.9%
12_BOS	4.69	100%	100%	100%	100%	100%	4.36	100%	100%	100%	100%	100%	68.13	100%	100%	100%	100%	100%
13_BOS	4.64	100%	100%	100%	100%	100%	3.48	100%	100%	100%	100%	100%	68.14	100%	100%	100%	100%	100%
14_BOS	4.23	100%	100%	100%	63.2%	100%	2.90	100%	100%	100%	0%	0%	68.76	100%	100%	100%	0%	61.2%
15_BOS	4.72	100%	100%	100%	100%	100%	3.17	100%	100%	100%	100%	100%	67.04	100%	100%	100%	100%	100%
16_BOS	4.41	100%	100%	100%	100%	100%	2.94	100%	100%	100%	100%	100%	68.82	100%	100%	100%	100%	100%
17_BOS	4.10	100%	100%	100%	100%	100%	4.71	100%	100%	100%	100%	100%	68.68	100%	100%	100%	100%	100%
18_BOS	4.48	100%	100%	100%	100%	100%	2.83	100%	100%	100%	100%	100%	67.76	100%	100%	100%	100%	100%
19_BOS	5.06	100%	100%	100%	100%	100%	3.16	100%	100%	100%	100%	100%	67.28	100%	100%	100%	100%	100%
